# Fabrication and Characterization of Celecoxib-Loaded Chitosan/Guar Gum-Based Hydrogel Beads

**DOI:** 10.3390/ph16040554

**Published:** 2023-04-06

**Authors:** Rukhsana Batool, Jahanzeb Mudassir, Mahtab Ahmad Khan, Saman Zafar, Sadia Jafar Rana, Nasir Abbas, Amjad Hussain, Muhammad Sohail Arshad, Sajjad Muhammad

**Affiliations:** 1 Faculty of Pharmacy, Bahauddin Zakariya University, Multan 60800, Pakistan; rukhsana.batool@tih.org.pk (R.B.);; 2 Faculty of Pharmaceutical Sciences, University of Central Punjab, Lahore 54000, Pakistan; 3 University College of Pharmacy, University of the Punjab, Lahore 54590, Pakistan; 4 Department of Neurosurgery, University of Helsinki and Helsinki University Hospital, 00290 Helsinki, Finland; 5 Department of Neurosurgery, Medical Faculty and University Hospital Düsseldorf, Heinrich-Heine-Universität Düsseldorf, 40225 Düsseldorf, Germany

**Keywords:** hydrogel beads, chitosan, guar gum, celecoxib, crosslinking, inflammation, inflammatory markers, sustained release, oral

## Abstract

The aim of this study was to fabricate celecoxib-loaded chitosan/guar gum (CS/GG) single (SC) and dual (DC) crosslinked hydrogel beads using the ionotropic gelation approach. The prepared formulations were evaluated for entrapment efficiency (EE%), loading efficiency (LE%), particle size and swelling studies. The performance efficiency was assessed by in vitro drug release, ex-vivo mucoadhesion, permeability, ex-in vivo swelling and in vivo anti-inflammatory studies. The EE% was found to be ~55% and ~44% for SC5 and DC5 beads, respectively. The LE% was ~11% and ~7% for SC5 and DC5 beads, respectively. The beads showed a matrix-like network with thick fibers. The particle size of beads ranged from ~2.74 to 1.91 mm. About 74% and 24% celecoxib was released from SC and DC hydrogel beads, respectively, within 24 h. The SC formulation showed higher %swelling and permeability than the DC counterpart, while the %mucoadhesion was relatively higher for DC beads. During the in vivo study, a significant decrease in the inflammation of the rat paw and inflammatory markers including C-reactive proteins (CRP) and interleukin-6 (IL-6) was observed following treatment with the prepared hydrogel beads; however, the SC formulation showed better therapeutic efficiency. In conclusion, celecoxib-loaded crosslinked CS/GG hydrogel beads can provide sustained drug release and act as potential candidates for managing inflammatory conditions.

## 1. Introduction

Celecoxib (CX), a selective cyclooxygenase-2 inhibitor non-steroidal anti-inflammatory drug (NSAID), is the drug of choice for chronic inflammatory diseases. However, its erratic absorption, reduced aqueous solubility and bioavailability require the administration of high doses (200–400 mg twice a day) to achieve the desired outcomes [1]. The long-term use of high doses results in serious gastrointestinal tract (GIT)-related side effects including bleeding, ulcerations, heart burn, discomfort, and perforations [1,2]. In order to overcome these problems, sustained release monolithic (single unit) and multi-particulate drug delivery systems have been developed. The monolithic system is associated with several limitations including variations in transit and residence times at various sites of GIT and undesired formulation disintegration, thus resulting in reduced bioavailability. Multi-particulate or multi-unit systems are advantageous over monolithic systems due to a reduced risk of toxicity, less inter- and intra-individual variability, a quick transit from upper GIT and a prolonged residence time in the colonic region. Some examples of these systems include microspheres, matrices, nanoparticles, pellets, hydrocolloids and hydrogel beads [3,4].

Hydrogel beads, multi-particulate (0.2–3.0 mm) polymeric systems, swell in response to aqueous environments and release drugs smartly. Hydrogel beads have been fabricated by using several approaches including ionotropic gelation, polyelectrolyte complexation, syringe dropping/extruding, air atomization, emulsion gelation, the incorporation of both oils and waxes and impinging technology. Among these, ionotropic gelation is the simplest and most convenient method. Several natural and synthetic polymers such as acrylic acid, guar gum, chitosan and sodium alginate can be used for preparing hydrogel beads. Among these materials, natural polymers, being biocompatible, non-toxic and renewable, may offer better drug delivery characteristics [5,6]. Such properties can be further modified by using different crosslinkers.

Guar gum (GG), a polysaccharide, is made up of sugars, galactose and mannose. Chitosan (CS), a cationic biodegradable and biocompatible polymer, comprises repeated units of (2-amino-2-deoxy-D-glucopyranose). GG and CS can serve as potential candidates for formulating hydrogel beads due to their release extending properties, pH responsiveness and susceptibility to degradation by the intestinal microbes [7,8].

Sodium tripolyphosphate (STPP or TPP) interacts with positively charged amino groups of CS via its negatively charged counter ions. Glutaraldehyde (GLA) forms imine bonds with amine groups of CS via its aldehyde groups [9].

The present work aimed to develop celecoxib-loaded hydrogel beads comprising GG and CS (as base materials) and TPP and GLA (as crosslinkers) by using the ionotropic gelation technique. The fabricated beads were evaluated for an in vitro drug release study, ex vivo swelling, mucoadhesion, a permeation study and an in vivo anti-inflammatory study in a rat paw oedema model.

## 2. Results

### 2.1. Preparation of Tripolyphosphate and Glutaraldehyde Crosslinked Chitosan/Guar Gum-Based Hydrogel Beads

**Figure 1:**
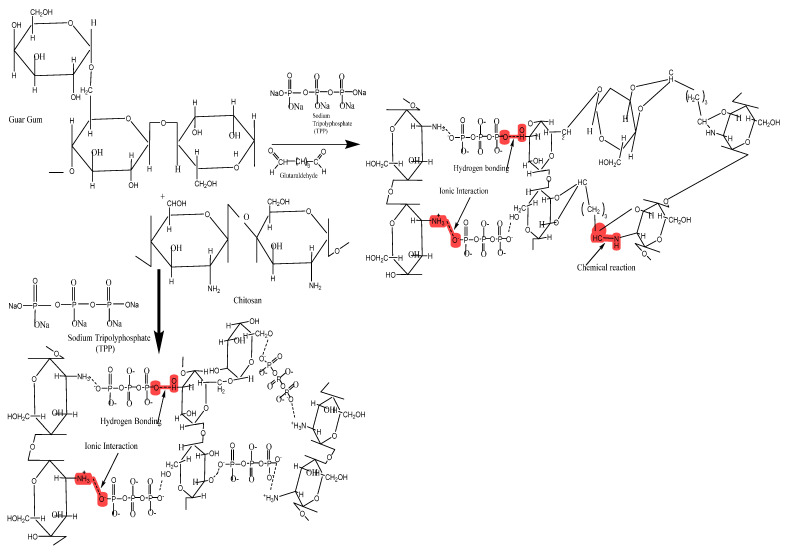
Hypothetical crosslinked structure of CS/GG-based SC and DC hydrogel beads.

A schematic diagram of interaction between polymers and crosslinkers is represented in Figure 1. Phosphate (PO_4_^−^) groups of TPP interact with protonated amino (NH_3_^+^) groups of CS in the acetic acid solution. TPP interacts with hydroxyl (-OH) groups of GG via hydrogen bonding, hence offering a complex linked structure [10]. Amine groups of CS interact with aldehyde groups of GLA, resulting in the formation of a stable imine bond [9]. GG also interacts with the aldehyde group of GLA.

### 2.2. Optimization of Single and Dual Crosslinked Hydrogel Beads

#### 2.2.1. Particle Size

**Figure 2:**
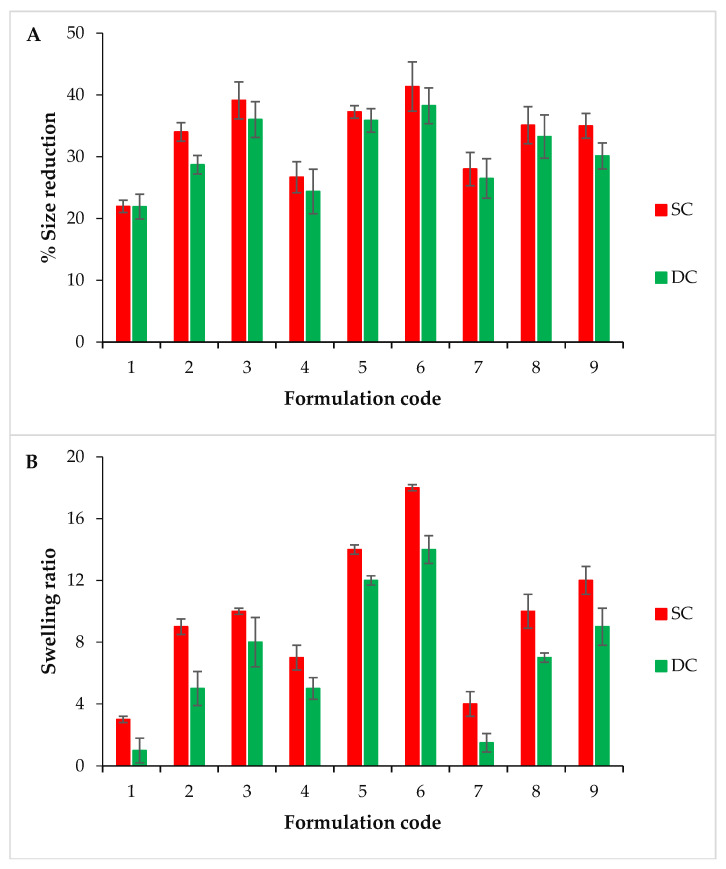

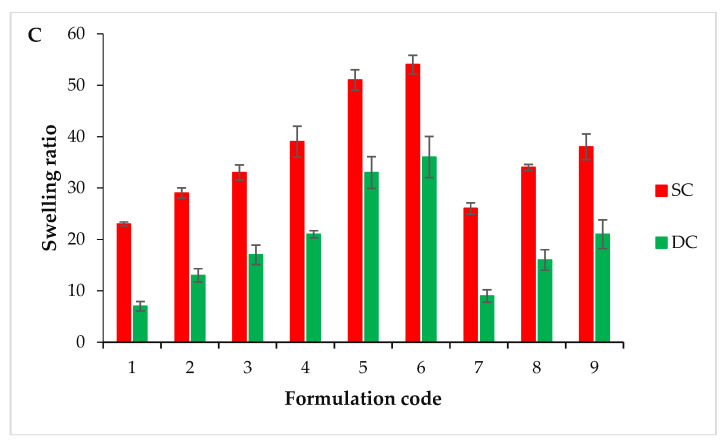
Comparison of the percentage size reduction (**A**), comparison of the swelling ratio at pH 1.2 (**B**) and pH 7.5 (**C**).

The particle size of all (L, M, H) wet SC beads ranged from 1.65 ± 0.018 mm to 3.64 ± 0.025 mm. For wet DC formulations (L, M, H), the particle size of all (L, M, H) wet DC formulations ranged from 1.28 ± 0.035 mm to 2.37 ± 0.013 mm. The DC beads showed a lower particle size as compared to the SC counterparts, possibly due to the high crosslinking density (thus, the more compact polymeric matrix) [11]. Dry SC formulations (L, M, H) showed a particle size ranging from 1.54 ± 0.017 mm to 2.74 ± 0.019 mm. In contrast, the particle size of dry DC beads (L, M, H) ranged from 1.2 ± 0.009 mm to 1.91 ± 0.007 mm. These results indicated that the particle size of DC beads in the dry state is lower than that of SC beads. The size reduction (%) of all DC beads was found to be less than that of the respective SC beads due to the lesser water holding capacity of their tightly packed structure (Figure 2A).

#### 2.2.2. Swelling Studies

The SC and DC beads showed (Figure 2B,C) an increased swelling ratio with an increasing GG content at both pH values (i.e., 1.2 and 7.5) for all L, M, and H samples. This increase in the swelling was possibly due to its hydrogen bonding with water and an increased hydrophilicity. The order of swelling for SC and DC beads was found as M ˃ H ˃ L at both pH values. In case of SC beads, the maximum swelling ratio was presented by SC6 (18 ± 0.43 at pH 1.2 and 54 ± 0.25 at pH 7.5) and SC5 (14 ± 0.29 at pH 1.2 and 51 ± 0.50 at pH 7.5) of the M category. In the case of DC beads, the maximum swelling ratio was observed for DC6 of M (14 ± 0.30 at pH 1.2 and 36 ± 0.38 at pH 7.5) and DC5 (12 ± 0.26 at pH 1.2 and 33 ± 0.29 at pH 7.5). This swelling behavior was due to the relatively greater strength and, thus, water upholding capacity of M beads (comprising 2% *w*/*v* CS) compared to that of L beads (composed of 1.5% *w*/*v* CS). As compared to M samples, the relatively more compact and tightly packed structure of H beads (containing 2.5% *w*/*v* CS) led to a relatively lower water uptake. AJ Sami et al. presented that the swelling ratio depends upon the amount of CS used [11].

All the formulations showed higher swelling ratios at pH 7.5 than at pH 1.2. The higher swelling ratio at a high pH was probably due to the degradation of galactan and mannan units by phosphate buffer ions. Moreover, the hydrophilicity of CS and electrostatic repulsion between functional groups were possibly responsible for the relatively loose/less compact structure. The same phenomenon was reported by K.M. Manjanna et al. for hydrogel mirobeads [12]. However, the swelling ratio was found to be greater for all SC beads than for the respective DC beads. A possible reason for this decreased swelling trend of the DC formulation was the relatively stronger interaction between the hydroxyl groups of GG and the amine groups of CS, thus leading to an increased crosslinking density and a more compact and tight packing of beads. These results are consistent with those presented by E Budianto et al., i.e., tightly packed GLA crosslinked CS hydrogels allowed less water uptake and a low swelling ratio [13].

#### 2.2.3. Optimization

**Figure 3:**
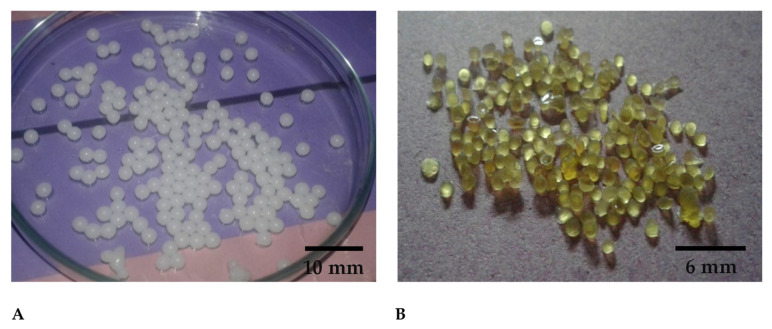
Digital images of optimized (**A**) Wet hydrogel beads, (**B**) Dry hydrogel beads.

M and H samples of both SC and DC beads exhibited appropriate particle sizes compared to L samples. The M samples showed a better swelling profile. Among the M samples, SC6 and DC6 presented good results in terms of swelling. Other characteristics such as the lesser shape uniformity, non-flexibility during reproduction (viscous solutions) and handling (sticky beads) and unstable nature during swelling were excluded while optimizing the best formulation. The swelling ratio of SC5 and DC5 (M) was not significantly different from that of SC6 and DC6 (M). Moreover, SC5 and DC5 presented good results regarding particle size, strength, shape uniformity and stability during relevant studies (Figure 3A,B). In the context of these results, M samples in general and SC5 and DC5 specifically were selected for further studies.

### 2.3. Entrapment and Loading Efficiency

The entrapment efficiency (EE%) was found to be 55.00 ± 0.028% and 43.75 ± 0.016% for SC5 and 43.75 ± 0.016% for DC5 beads, respectively. The loading efficiency (LE%) was found to be 11.00 ± 0.030% and 7 ± 0.018% for SC5 and DC5 beads, respectively. As the results indicate, LE% and EE% were higher for SC beads as compared to DC beads. A possible reason includes the decreased distance between molecules due to the high crosslinking density, which permitted a low amount of the drug to be entrapped within the DC hydrogel beads. Natalia Sedyakina et al. also reported that a high crosslinking density reduced the entrapment efficiency of bovine serum albumin in the polymer matrix [14].

### 2.4. Scanning Electron Microscopy

**Figure 4:**
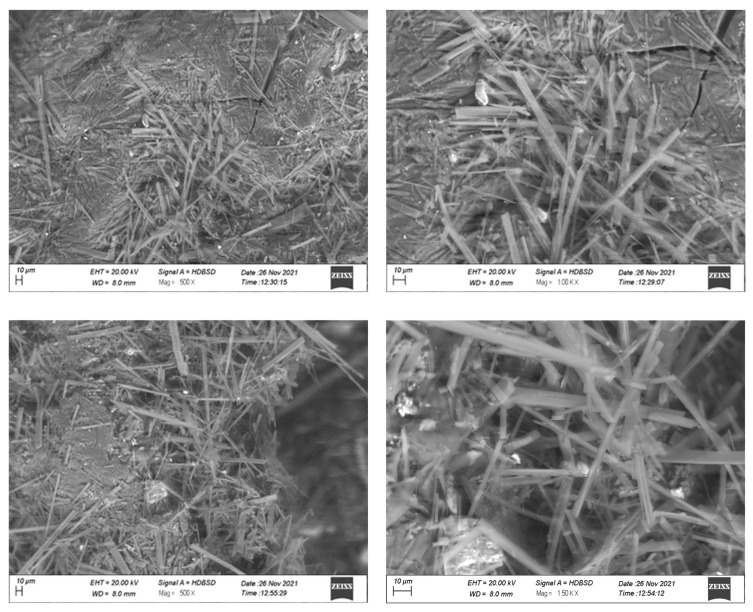
SEM images of DC5 (**top**) and SC5 (**bottom**) hydrogel beads.

Scanning electron microscopy (SEM) images (Figure 4) revealed a matrix-like network with thick fibers in the case of drug-loaded SC5 hydrogel beads. The porous structure of the beads possibly merged to form a matrix. SEM images of both hydrogel bead formulations, i.e., SC5 and DC5, suggested that the beads are capable of holding a large amount of water (SC5 having a relatively greater capacity) [9].

### 2.5. Fourier Transform Infrared Spectroscopy

**Figure 5:**
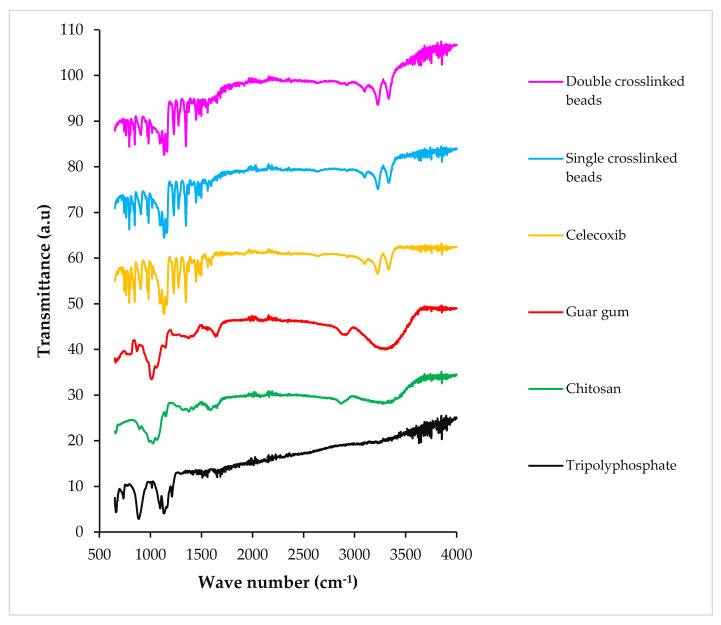
FTIR spectra of TPP (sodium tripolyphosphate), CS (chitosan), GG (guar gum), CX (celecoxib), SC and DC hydrogel beads.

The Fourier transform infrared (FTIR) spectrum of CS displayed a characteristic broad band between 3200 cm^−1^ and 3400 cm^−1^ due to -OH stretching vibrations and the presence of the -NH_2_ group, respectively. The bands at 2875 cm^−1^, 1374.04 cm^−1^, 1647.50 cm^−1^ and 1023.42 cm^−1^ represented -C-H, -C-N and -C=O stretching vibration (amide group) and presence of -C–O, respectively, thus, representing the polysaccharide structure of chitosan [9,15]. GG displayed a broad band between 3250 cm^−1^ and 3400 cm^−1^ due to -OH stretching vibrations. The peaks between 3000 cm^−1^ and 2800 cm^−1^ represented -CH stretching bonds [16]. The CX spectrum displayed peaks at 3340 cm^−1^, 1348 cm^−1^ and 1165 cm^−1^, which were assigned to NH, asymmetric SO_2_ and symmetric SO_2_ stretching vibrations, respectively [17]. TPP showed an intense band around 3600 cm^−1^ representing its free -OH groups. The optimized SC5 beads showed spectral peaks from 2800 cm^−1^ to 3400 cm^−1^, indicating the reaction between TPP and amine groups [18]. DC5 beads showed a small increase in band intensity at 2850 cm^−1^, depicting the overlapping of the -C-H bond with -CH_2_- groups of GLA. The stretch band at 1646 cm^−1^ related to the imine bond represented the crosslinking reaction between GLA and CS amino groups. The CX-specific peaks at 3340 cm^−1^, 1348 cm^−1^ and 1165 cm^−1^ appeared in both SC5 and DC5 beads. Therefore, the observation confirmed the successful loading of CX in SC5 and DC5 beads (Figure 5).

### 2.6. X-ray Diffraction

**Figure 6:**
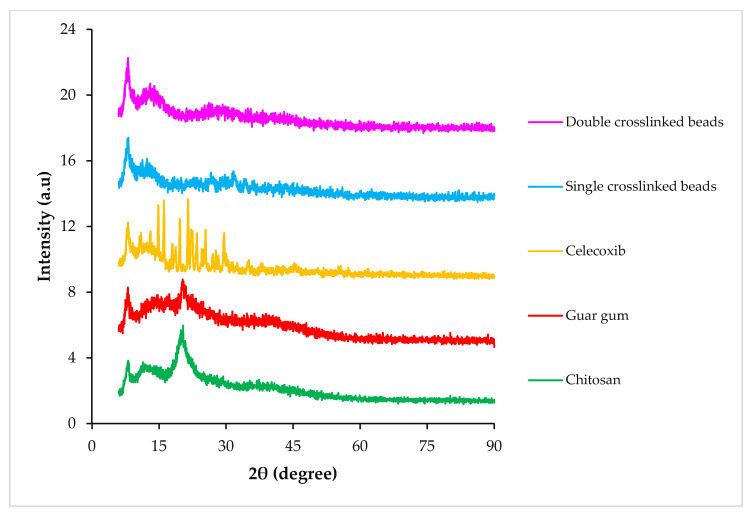
XRD scans of CS (chitosan), GG (guar gum), CX (celecoxib), SC and DC hydrogel beads.

The X-ray diffraction (XRD) scan of CS showed a characteristic peak at 2*θ* = 19.94°, representing its crystalline structure [19]. Pure GG showed a low-intensity broad peak around 20.94°, which represented its amorphous nature [9]. CX exhibited intense and long peaks at 2*θ* = 11.0°, 14.84°, 16.08°, 21.52° and 29.6°, confirming its crystalline nature. The SC5 and DC5 beads showed less intense peaks, indicating a considerable decrease in the crystallinity of the drug following incorporation within the polymeric hydrogel beads (Figure 6).

### 2.7. Thermal Analysis

**Figure 7:**
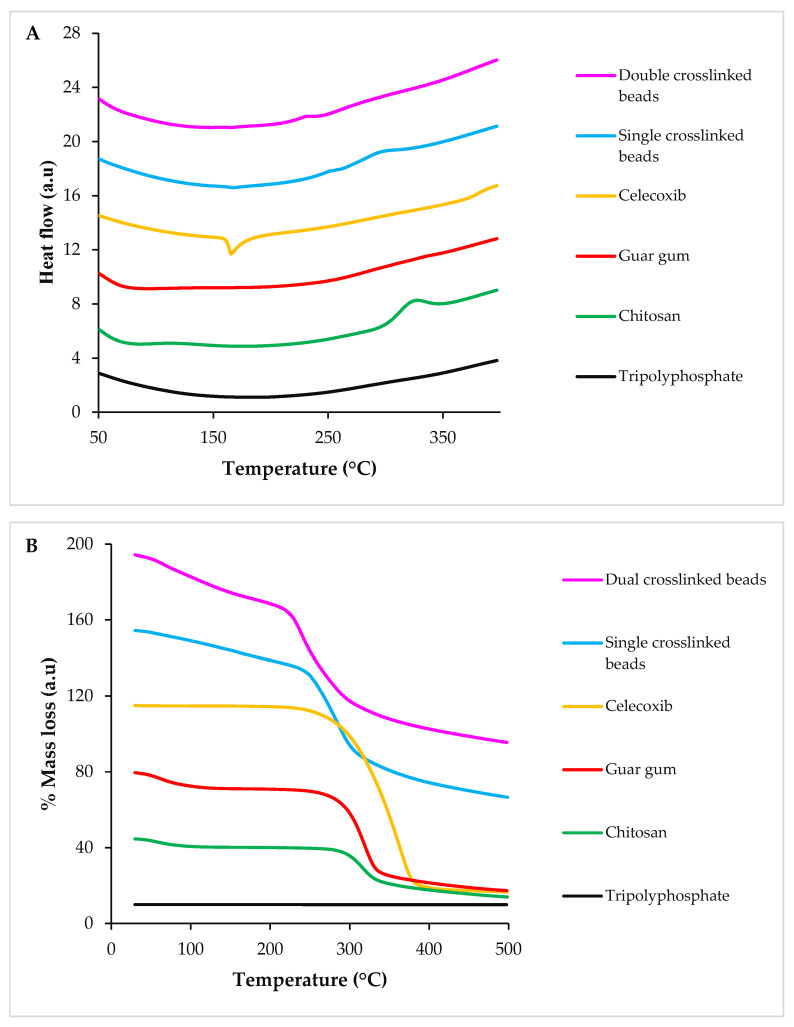
DSC (**A**) and TGA (**B**) scans of TPP (sodium tripolyphosphate), CS (chitosan), GG (guar gum), CX (celecoxib), SC and DC hydrogel beads.

The differential scanning calorimetry (DSC) thermogram of CS manifested a shallow, broad endotherm around 90 °C, relating to dehydration. A more prominent exothermic peak at 280 °C was attributed to the thermal decomposition of the polymeric material [20]. GG showed an endothermic transition at ~78 °C, suggesting its dehydration [21]. CX showed a characteristic endothermic peak at 165 °C, which was related to its melting. The DSC thermogram of TPP did not show any thermal transition until 500 °C, suggesting its high thermal stability. The drug-loaded crosslinked beads showed a high thermal stability, as any transition was not observed until ~230 °C. An exotherm around 300 °C in the DSC scan of the SC formulation suggested its degradation. In the case of DC hydrogel beads, a transition around 230–250 °C suggested its degradation (Figure 7A).

The TGA (thermogravimetric analysis) thermogram of CS showed approximately 10% weight loss until 100 °C due to the removal of adsorbed moisture. Another weight loss of 45% was observed at 270 °C because of the thermal decomposition of the polymer [10,22]. The thermogram of GG displayed 12% weight loss until 120 °C due to the evaporation of adsorbed water. Around 50% weight loss was observed over a temperature range of 217–307 °C, which indicated thermal degradation. The TGA scan of CX manifested one step decrease (85%) in the mass of the sample over a temperature range of 248–382 °C. The TGA scan of TPP did not show any mass loss. Drug-loaded SC hydrogel beads showed around a 13% decrease in mass until 215 °C, which suggested the evaporation of water. A sharp decrease (i.e., 33%) in mass was observed from 230 °C to 315 °C, which suggested the thermal decomposition of the fabricated beads. Drug-loaded DC beads reflected a 15% weight loss until 210 °C, which manifested dehydration. From 212 °C to 312 °C, an additional 30% weight loss was recorded, indicating its thermal decomposition (Figure 7B). These results suggested that fabricated hydrogel beads are thermally stable.

### 2.8. In Vitro Drug Release Studies

**Figure 8:**
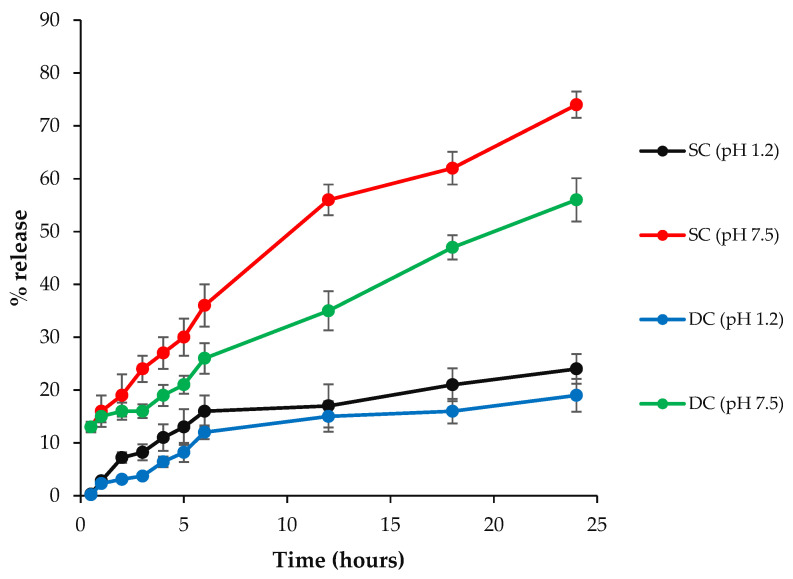
% Release of the drug from SC5 and DC5 beads at pH values of 7.5 and 1.2 at specified time intervals.

The results indicated that there is a direct relation between the swelling pattern and release of a drug from the hydrogel beads. Figure 8 revealed that, at pH 7.5, SC5 showed a slow drug release for the first 6 h. An increase was observed in the release of the drug for the next 6 h. Maximum (74%) drug release was observed at the 24th hour. Similar behavior was observed at pH 1.2, with the exception that a very slow increase in the release was observed, reaching up to a maximum of 24.1% at the 24th hour. DC5 showed a slight increase in the %release for the first 5 h, and then a controlled release was observed until the 24th hour at both pH values. The value of R^2^ for both SC5 (0.939 at pH 1.2 and 0.986 at pH 7.5) and DC5 (0.915 at pH 1.2 and 0.961 at pH 7.5) beads depicted that the drug release profile followed the Korsmeyer Peppas model, with SC5 values closer to one at both pH values. The values of ‘n’ for SC5 (0.502 at pH 1.2 and 0.525 at pH 7.5) and DC5 (0.621 at pH 1.2 and 0.520 at pH 7.5) revealed that the beads follow non-Fickian diffusion.

### 2.9. Ex Vivo Studies

#### 2.9.1. Mucoadhesion studies

The percentage of beads that remained adhered to the goat intestinal mucosa was 0 ± 0% and 8 ± 0.82% for SC and DC beads, respectively, after 24 h (Table 1). The number of beads detached from the mucous membrane was higher in the case of SC5 beads as compared to in the case of DC5 beads. This indicated the relatively strong mucoadhesion of the DC5 formulation with excised goat intestinal mucosa.

#### 2.9.2. Permeability Studies

**Figure 9:**
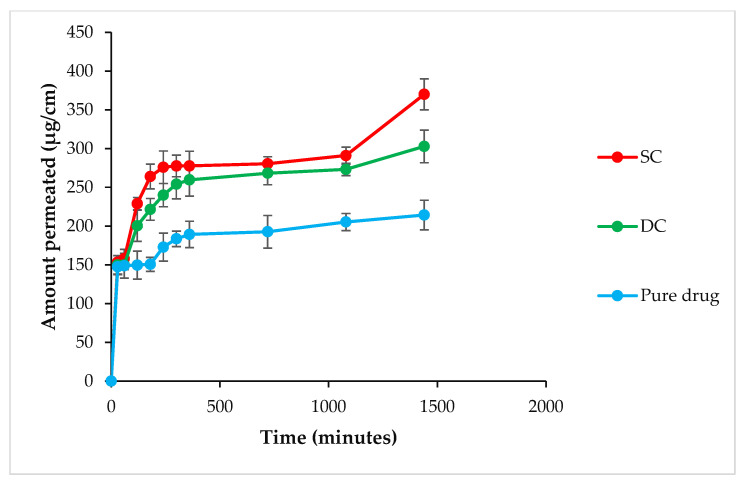
Amount of the drug permeated across the intestinal mucosa in the case of the pure drug and SC and DC beads.

The pure CX, SC5 and DC5 beads were compared for Papp (apparent permeability coefficient). The Papp value for pure CX was lower (0.332 × 10^−3^ cm/min) as compared to that observed for SC5 (0.733 × 10^−3^ cm/min) and DC5 beads (0.643 × 10^−3^ cm/min). The amount of CX that permeated through the intestinal mucosa was higher in the case of prepared hydrogel beads when compared to the pure drug (Figure 9), possibly due to an improved residence time of CX after incorporation into the polymeric formulation. Moreover, an interaction between the polymers and intestinal cells possibly led to an increased mucoadhesion time and thus permeation of the drug from the fabricated beads [23,24,25]. As compared to DC, a higher flux from the SC formulation indicated its relatively lower or weaker retarding property [24].

#### 2.9.3. Swelling Studies

**Figure 10:**
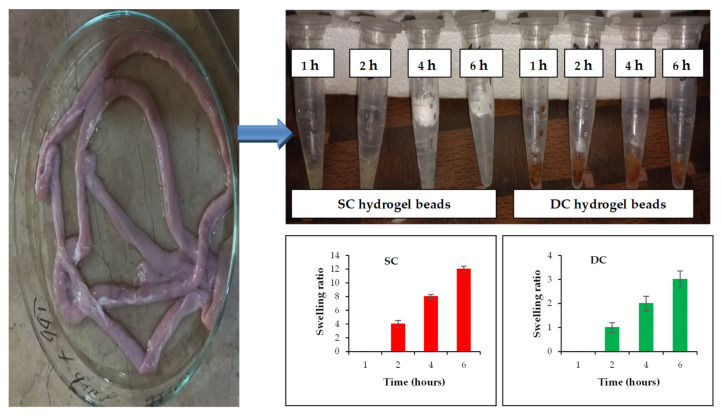
Ex-in vivo swelling behavior of optimized hydrogel beads (SC and DC) collected from excised rabbit organs at different time intervals.

In excised organs (stomach and intestine), orally administered formulations, i.e., SC5 and DC5 beads, remained intact in the 1st hour. After 2 h, the SC5 beads displayed swelling to a certain extent, while the DC5 beads showed negligible swelling. After 4 h, the SC5 beads showed significant swelling, while the DC5 beads did not show further swelling. After 6 h, the SC5 beads started to erode due to excessive swelling, while the DC5 beads were still intact (Figure 10). The excessive water uptake by the SC5 beads was possibly due to the relatively more porous structure, which resulted in a higher degree of swelling and subsequent erosion [26].

### 2.10. In Vivo Anti-Inflammatory Activity

**Figure 11:**
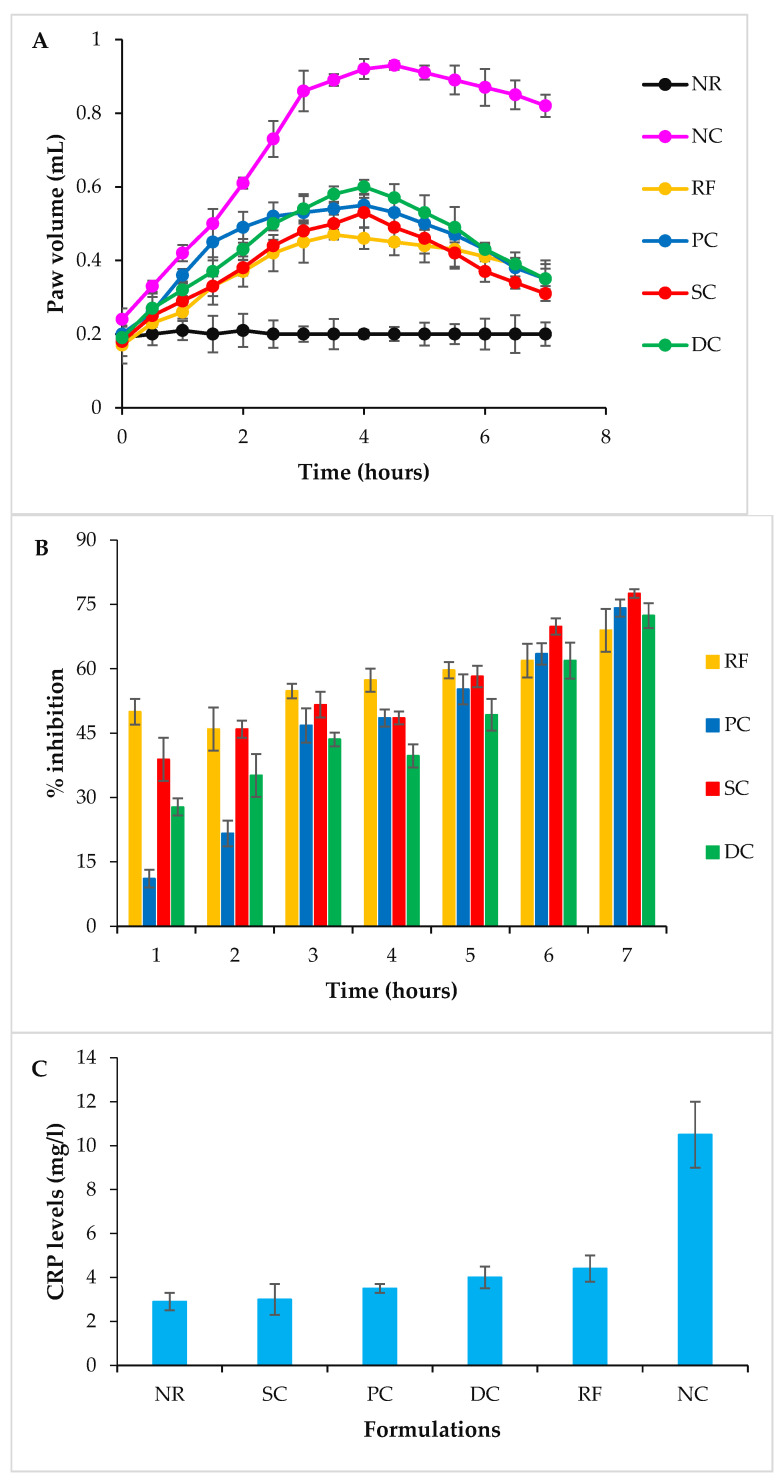

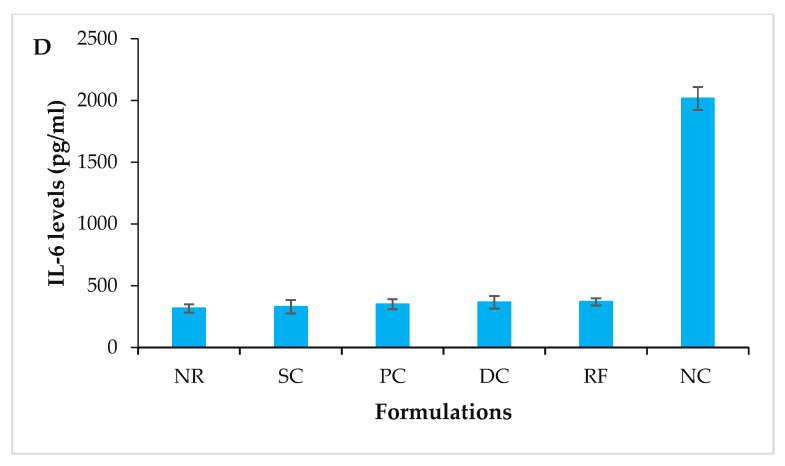
Anti-inflammatory activity using the carrageenan-induced rat paw edema model (**A**) paw volume (mL) in different hours. Data show the mean ± SD and were statistically analyzed by two-way ANOVA followed by Dunnett’s test (*p* ˃ 0.05). (**B**) % Inhibition at different hours. Data show the mean ± SD and were statistically analyzed by two-way ANOVA followed by Dunnett’s multiple comparison test using GraphPAD Prism (version 6.0). (**C**) Serum CRP levels of various rat groups 7 h after CG injection; mean ± S.D. (**D**) Serum IL-6 levels of various rat groups 7 h after CG injection.

The induction of oedema was confirmed by measuring the increase in the paw volume using a pleythysmometer. The reduction in paw volume was considered as an improvement of the disease state, which was interpreted by calculating the percentage of oedema inhibition. The results indicated that the paw volume of all groups increased after carrageenan (CG) administration, except for the NR (normal) group. In the case of the NC (negative control) group, the maximum paw volume of 0.92 mL was observed at the 4th hour after CG injection, which was subsequently reduced to 0.82 mL at the 7th hour. The RF (reference) group showed a maximum paw volume of 0.47 mL at the 4th hour, which was subsequently reduced to 0.35 mL at the 7th hour. The PC (positive control) group showed a maximum paw volume of 0.48 mL at the 4th hour, which was subsequently reduced to 0.37 mL at the 7th hour. The SC (single crosslinked) and DC (double crosslinked) beads-administered groups showed maximum paw volumes of 0.55 mL and 0.65 mL, respectively, at the 4th hour, which were subsequently reduced at the 7th hour, i.e., 0.37 mL and 0.44 mL, respectively (Figure 11A). The SC and DC groups showed a slow increase and then a decrease in the paw volume. There was a negligible variation in the paw volume of the NR group throughout the study. The SC group showed better results than all treated groups. The results indicated a significant decrease in the paw volumes of the SC and DC groups when compared to the RF and PC counterparts; therefore, it can be concluded that the fabricated polymeric beads started to degrade after emptying from the stomach and reaching the higher pH environment due to their pH-sensitive nature. The % inhibition of inflammation exhibited the order of SC > PC > DC > RF > NC (Figure 11B). SC showed better results than all the treated groups [27,28].

#### 2.10.1. Determination of Inflammatory Markers

The C-Reactive protein (CRP) levels of the NR, SC, PC, DC, RF and NC groups were found to be 2.9 mg/L, 3.0 mg/L, 3.5 mg/L, 4 mg/L, 4.4 mg/L and 10.5 mg/L, respectively (Figure 11C). In the NC group, the levels of inflammatory markers increased significantly. The results revealed that all the treatment groups exhibited significantly reduced serum levels of CRP. However, SC showed the lowest levels of CRP, indicating their efficiency in decreasing inflammation. The interleukin-6 (IL-6) levels of the NR, SC, PC, DC, RF and NC groups were found to be 316 pg/mL, 330 pg/mL, 350 pg/mL, 366 pg/mL, 370 pg/mL and 2016 pg/mL, respectively (Figure 11D). All treatment groups represented significantly reduced levels of IL6; however, SC showed the lowest level of IL-6. The reason behind this decrease was the release of a sufficient amount of the drug that reduced the levels of acute inflammatory markers i.e., CRP and IL-6. These effects were in accordance with the already reported work [29].

#### 2.10.2. Histopathological Examination of Paw Tissues

**Figure 12:**
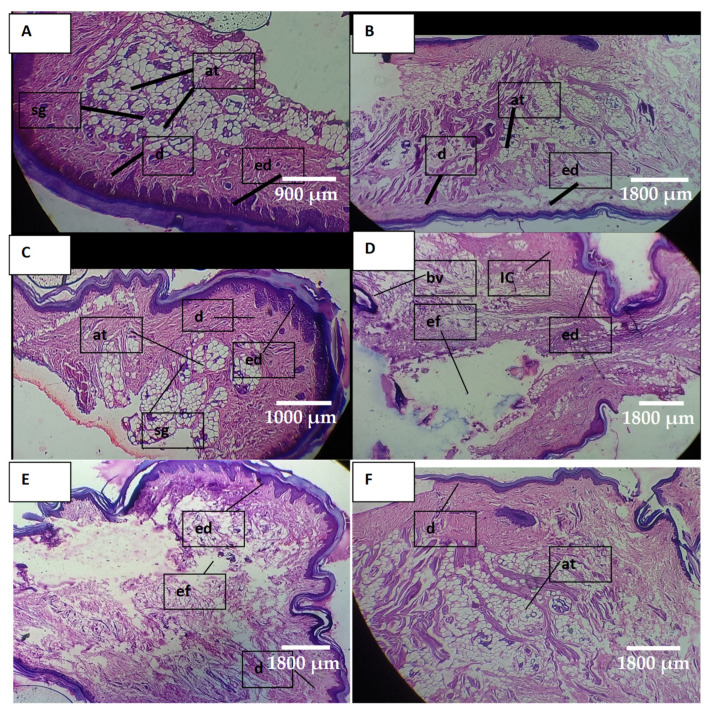
Photomicrograph of paw tissue: (**A**) NR group, (**B**) PC group, (**C**) RF group, (**D**) NC group, (**E**) SC group, (**F**) DC group, where ed = epidermis, d = dermis, at = adipose tissue, sg = sebaceous glands, IC = inflammatory cells, bv = blood vessel and ed = edematous fluid.

Histological images of the NR group (Figure 12A) depicted normal epidermis, dermis and adipose tissues. The NC group (Figure 12D) exhibited severe inflammation of the epidermis and a huge amount of edematous fluid. The blood vessels were entrapped within the inflamed cells. A reduction in inflammatory cells was observed in the PC and RF groups (Figure 12B,C, respectively) Moreover, the epidermis was regenerated, and sebaceous glands were visible in the tissue. The PC group showed a significantly improved (in terms of inflammation) epidermis and dermis. The SC group (Figure 12E) showed a significant decrease in inflammatory cells and a regenerated dermis. The DC group (Figure 12F) exhibited a regenerated epidermis, less inflamed cells and a low amount of edematous fluid. Both the SC and DC groups showed a reduction in inflammation in a controlled manner. However, SC showed relatively better results, comparable with the RF and PC groups. The above results were consistent with the previous reports in the literature [30].

## 3. Discussion

The oral route is the most preferred option for drug delivery due to the ease of administration and the good patient compliance [31]. However, some limitations are associated with the oral route such as first pass metabolism, a short retention time in the GIT and low bioavailability. During recent years, hydrogels have emerged as potential candidates for oral delivery because of their biocompatibility, their biodegradability, their easy fabrication, their ability to protect the drug from harsh GIT conditions, the increased residence time of the drug within the GIT and their ability to provide the release of the drug at a desired rate [1]. Celecoxib, commonly administered via the oral route, is one of the most widely used anti-inflammatory agents. However, the long-term use of this NSAID leads to GIT-relevant side effects such as bleeding, ulceration, discomfort and perforation [1,2]. The encapsulation of celecoxib in a multi-particulate hydrogel beads formulation can overcome the above-stated drawbacks and lead to an efficient drug delivery. Multi-particulate systems exhibit a prolonged residence time in the colon, which can serve as an absorbing organ and permit the absorption and entry of drugs to the general circulation with a high bioactivity due to the avoidance of drug degradation in harsh conditions of the upper GIT [32].

In the present study, single (SC, TPP) and dual (DC, TPP and GLA) crosslinked CS- and GG-based hydrogel beads incorporated with celecoxib were prepared by using the ionotropic gelation technique. CS is a physiologically compatible, naturally occurring polysaccharide with mucoadhesive properties (due to the strong electrostatic interaction between the positively charged amino groups of CS and negatively charged sialic acid found on the mucus surface and hydrophilic as well as hydrophobic interactions). Moreover, CS is degraded/metabolized by the enzymes released by colonic microbes, particularly N-acetyl-β-glucosaminidase, leading to the release of encapsulated active ingredient in the colon [32]. Likewise, naturally occurring biodegradable, non-toxic GG is degraded by colon-inhabiting Bacteroides and Ruminococcus; thus, it can serve as an efficient drug carrier for oral delivery [33]. TPP and GLA have been widely employed for crosslinking polymeric materials in order to attain sustained release characteristics [9].

Dry DC beads displayed a relatively small particle size (i.e., ~1.2–1.91 mm) as compared to the SC formulation (i.e., ~1.54–2.74 mm). Swelling studies were performed at pH 1.2 and 7.5, relating to the pH of the stomach and ileo-colonic region in a fasting state, respectively. The ileo-colonic region offers a high retention time for orally administered beads and thus improves the drug absorption. Moreover, higher pH value as well as abundant microbial flora in this region significantly contribute towards the degradation of polymers (e.g., CS and GG in the present study) and thus favor the release of the loaded drug [7,34]. DC formulations showed a relatively lower percentage of swelling than SC beads due to the closely packed structure governed by a high crosslinking density. The fabricated DC hydrogel beads showed relatively lower EE% and LE% (i.e., ~43% and 7%, respectively) when compared to the SC counterpart (i.e., ~55% and ~11%, respectively). A possible reason for the relatively reduced size, low EE% and LE% was the high crosslinking density, which resulted in a more compact formulation matrix and permitted a low amount of celecoxib to be encapsulated in the DC formulation. The FTIR spectra of the prepared formulations displayed peaks of celecoxib, suggesting the successful incorporation of drug within the hydrogel beads. The DSC and XRD results indicated that the active ingredient was encapsulated in an amorphous state within the beads. The TGA results suggested the high thermal stability of celecoxib-loaded hydrogel beads. SEM images revealed that the beads exhibited a matrix-like porous structure, which suggested that the physiological fluid would easily penetrate into the fabricated formulation and result in the release of the drug. During the in vitro release study, celecoxib was released from fabricated beads in a sustained manner. The SC formulation released ~74% of the drug within 24 h, while only 24% of the drug was released in the case of DC hydrogel beads. The higher crosslinking in the DC formulation led to a more sustained release of the drug. Similarly, during the ex vivo permeability study, the drug permeation across the intestinal mucosa was higher in the case of the SC formulation because of the relatively loose and less compact structure. The DC formulation showed relatively more adherence to the intestinal mucosa than the SC formulation. The results of the ex-in vivo swelling study indicated that the SC formulation takes up more fluid when compared to the DC beads due to its relatively loose network. Likewise, treatment with the SC formulation led to a relatively higher reduction in the inflammation of the rat paw as compared to the DC formulation due to the higher %swelling, release and permeability. Nevertheless, both of the prepared formulations can be reliably used for the safe and sustained delivery of NSAIDs and the management of inflammatory conditions.

## 4. Materials and Methods

### 4.1. Materials

CS (low molecular weight (MW), ≥75% deacetylated), GG (MW 967,000 g/mol), TPP (technical grade 85%, MW 367.86 g/mole), GLA (grade II 25% in water, MW 100.12 g/mol), CG, dipotassium hydrogen phosphate and sodium chloride were purchased from Sigma Aldrich, Steinheim, Germany. CX was kindly donated by Getz Pharma Private Limited, Karachi, Pakistan. Potassium phosphate monobasic was purchased from Duksan, Ansan, South Korea. Distilled water of an in-house facility (Department of Pharmaceutics, Faculty of Pharmacy, Bahauddin Zakariya University, Multan, Pakistan) was used throughout the study. All other materials used were of analytical grade.

### 4.2. Preparation of Sodium Tripolyphosphate and Glutaraldehyde Crosslinked Chitosan/Guar Gum-Based Hydrogel Beads

Two types of formulations were prepared by using the ionotropic gelation technique [35], including (a) SC and (b) DC chitosan/guar gum-based hydrogel beads. The detailed method that was developed for the preparation of hydrogel beads is given below.

#### 4.2.1. Preparation of Single Crosslinked Hydrogel Beads

For the preparation of SC hydrogel beads, CS (1.5–2.5% *w*/*v*) was dissolved in 1.5% *v*/*v* acetic acid solution with stirring at 3000 rpm at room temperature, i.e., 25 ± 3 °C (labelled as solution A). The GG at the concentration ranges of 0.15–1.25% *w*/*v* was dissolved in distilled water under stirring at 80 ± 5 °C (labeled as solution B). Solutions A and B were mixed at 40 ± 3 °C with continuous stirring at 2000 rpm. The homogenous mixture was added drop-wise into 1% *w*/*v* TPP solution using a 21G needle connected to a 20 cc syringe under constant stirring. The formed beads were gently stirred for 6 h, which ensured complete crosslinking and hardening at 2000 rpm and 25 ± 3 °C temperature. The beads were filtered, washed with distilled water for the complete removal of the unreacted polymers and crosslinker, dried at room temperature and stored in a moisture-free environment for further use (part A).

#### 4.2.2. Preparation of Dual Crosslinked Hydrogel Beads

DC hydrogel beads were prepared by immersing the beads formed in Part A into 10 mL of 2% *w*/*w* (with respect to the dry mass of the polymers) GLA for 1 h. The beads were allowed to cure overnight, filtered, washed with distilled water, dried at 25 ± 3 °C and stored in air-tight containers for further analysis (Part B).

### 4.3. Preparation of Celecoxi-Loaded Hydrogel Beads

The drug was loaded in the prepared hydrogel beads by using the sequential method (active loading technique). Optimized wet SC and DC beads (70 mg each) were individually immersed into a hydroalcoholic solution (three parts water and three parts ethanol) of CX (0.5% *w*/*v*) and gently stirred for 24 h. The hydrogel beads were filtered, washed with distilled water to remove any free drug from the surface and dried at room temperature.

### 4.4. Optimization of Single and Dual Crosslinked Hydrogel Beads

Various formulations, represented in Table 2, were prepared for the optimization of SC and DC hydrogel beads.

#### 4.4.1. Particle Size

The SC and DC beads were dried at room temperature for 24 h (until the attainment of a constant weight). The sizes of the wet and dry beads were analyzed by using a measuring scale graduated in centimeters. The sizes were also determined by using an optical microscope equipped with a pre-calibrated optical micrometer. After complete drying, the %size reduction of all formulations was calculated by using the following formula.

%Size reduction = initial size − final size/initial size(1)

#### 4.4.2. Swelling Studies

Swelling studies were carried out at pH 1.2 and 7.5 buffer media that relate to the stomach and intestine, respectively. The dried hydrogel beads were weighed (70 mg) and immersed in 30 mL of buffer media at room temperature. The swollen hydrogel beads were removed at discrete time intervals, weighed and re-immersed in the same buffer solutions. The equilibrium swelling ratio, S(EQ), was determined with the help of the following equation.

S(EQ) = Wh/Wd(2)

where Wh represents the weight of swollen beads at equilibrium and Wd represents the initial weight of the dry beads.

#### 4.4.3. Experimental Design

General factorial 2^3^ design was applied for the comparison of the swelling ratio and size reduction [36]. Input variables (X1 = CS and X2 = GG) were varied at three levels (X1 = CS = 1.5, 2 and 2.5 and X2 = GG = 10, 30 and 50). The results were evaluated by Design Expert (version 7.0). The values assigned were 10 = −1, 30 = 0 and 50 = +1.

### 4.5. Entrapment and Loading Efficiency

A known amount of CX containing SC and DC hydrogel beads was immersed individually in 200 mL of 0.75% *v*/*v* acetic acid, followed by sonication. The solution was filtered, and the content of CX was determined by using ultraviolet-visible spectrophotometric analysis at λmax 252 nm. EE% and LE% were calculated by using the following equations [37].

EE% = At − Au/At × 100(3)

LE% = Ae/Ah × 100(4)

where At represents the total amount of the drug added to the hydrogel beads, Au represents the total amount of the un-entrapped drug, Ae represents the total amount of the entrapped drug and Ah represents the total weight of hydrogel beads (drug + polymer).

### 4.6. Scanning Electron Microscopy

SEM (Zeiss, Baden-Württemberg, Germany) was performed to study the surface morphologies of fabricated hydrogel beads (SC and DC). The samples were placed on carbon stubs, coated with graphene, and images were taken at various magnifications (500× to 1500×) and 20.00 kV.

### 4.7. Fourier Transform Infrared Spectroscopy

FTIR spectroscopy was performed to determine the drug–polymer interaction, polymer/crosslinker crosslinking and functional groups. Individual constituents and fabricated beads were placed on the stage of the spectrophotometer (ALPHA II, Bruker, NJ, USA), and the spectra were obtained over a wave number range of 650–4000 cm^−1^.

### 4.8. X-ray Diffraction

XRD analysis was performed to assess the phase of the drug, polymers and fabricated hydrogel beads. The samples were placed in the holder and scanned at a 2*θ* range of 06–90° and a step size of 0.04°, with the help of a diffractometer (D8 Discover, Bruker, NJ, USA).

### 4.9. Thermal Analysis

DSC and TGA (TA instruments, NC, USA) were used to determine the thermal stability of the pure ingredients and prepared beads. For DSC analysis, the samples (i.e., pure ingredients and fabricated beads) were added to alumina pans and heated from 50 to 400 °C at a rate of 10 °C/minute, and enthalpic changes were recorded. The calorimeter was calibrated for temperature and heat flow under nitrogen purging (50 mL/min) by using nickel as the standard. For TGA, the samples were heated from 30 to 500 °C, and the % weight/mass loss was determined.

### 4.10. In Vitro Drug Release Studies

A known amount of optimized hydrogel beads was added into a dialysis bag and placed into a type 1 dissolution apparatus containing 900 mL of buffer media of two different pH values (i.e., 1.2 and 7.5) at 37 °C and 100 rpm. After predetermined time intervals, a 5 mL sample was withdrawn, and the same volume of the buffer was added to maintain sink conditions. The concentration of the drug was measured by ultraviolet-visible spectrophotometric analysis at a λmax of 252 nm.

### 4.11. Ex Vivo Studies

The studies involving animals were approved by the ethical committee of Bahauddin Zakariya University, Multan, Pakistan (letter no. 213/PEC/2021).

#### 4.11.1. Mucoadhesion Studies

Mucoadhesion studies of optimized hydrogel bead formulations were performed by using the wash-off method, with a few modifications. The goat intestine (obtained from a local slaughterhouse) was cut into two pieces. Two sections of the mucosal membrane (dimensions 3 × 3 cm) were stored in normal saline. Mucosal membranes were individually placed in a petri dish, and a known mass of SC and DC beads (70 mg each) was added. An assembly was maintained in order to perform the experiment. The mounted tissues were suspended into a beaker containing 100 mL of phosphate buffered saline (PBS, pH 7.5) maintained at 37 °C and agitated at 100 rpm. The beads that shed off at specified time intervals were collected and weighed. The beads that remained attached to the mucosal membrane indicated %mucoadhesion [38]. The %mucoadhesion with respect to time was measured by using the following formula.

%Mucoadhesion = ma − md/ma(5)

where ma represents the total mass of beads and md represents the mass of beads that were shed off.

#### 4.11.2. Permeability Studies

The goat intestinal mucosa was sliced longitudinally into three equal segments of 4 × 2 cm^2^, rinsed with Krebs solution and tied into a sac [38]. The pouches were filled with pure drug and optimized beads formulations containing a known amount of CX. Both ends of the sacs were closed. The mucosal side was kept inside and the serosal side outside. The sacs were suspended in a beaker containing 100 mL of Krebs solution maintained at 37 °C and 50 rpm. An aliquot of 1.5 mL was drawn at discrete time intervals (0.5, 1, 2, 3, 4, 5, 6, 8, 12, 18 and 24 h), and the medium was replenished with the same volume of Krebs solution each time. The drug content was determined by using an ultraviolet-visible spectrophotometer at λmax 252 nm. The Papp was calculated by using the following equation.

Papp = F/A × Co cm/min(6)

where F = permeation flux (µg per minute), A = surface area of the intestinal membrane and Co = initial amount of the drug in the intestinal segment. The value of “F” can be calculated from the slope of the linear portion of the plot obtained from the cumulative amount of the drug permeated through the intestinal sac (µg/min).

#### 4.11.3. Swelling Studies

In this study, eight healthy rabbits were used. These rabbits were divided into two groups, i.e., A and B, each comprising four rabbits. The rabbits were not given access to food for 12 h; however, they were allowed to have free access to water. Groups A and B were administered with optimized SC and DC beads, respectively. The rabbits in each group were sacrificed at 1, 2, 4 and 6 h. The stomach and the intestine were removed, and the ex-in vivo swelling behavior of the beads was observed.

### 4.12. In Vivo Anti-Inflammatory Activity

#### 4.12.1. Induction of Carrageenan-Induced Rat Paw Oedema

Anti-inflammatory activity was performed by using a CG-induced rat paw oedema model [4], in which the disease state was interpreted by determining inflammatory markers and % oedema inhibition. The oedema was induced in rats by injecting 1% *w*/*v* CG solution (0.1 mL) into the plantar region of the right hind paw.

#### 4.12.2. Study Design

The animals were weighed (average weight 200 ± 6.3 g) and randomly divided into six groups labeled as NR, NC, RF, PC, SC and DC beads-treated (Table 3). After 30 min of the oral administration of doses (15 mg/kg), the sub-plantar injection of CG was administered in all groups (except NR). The paw volume of all rats was measured using a plethysmometer (Ugo Basile, Gemonio, Italy) 0.5 h before and 1, 2, 3, 4, 5, 6 and 7 h after CG injection. In preliminary studies, it was observed that the disease state starts to develop within 1–2 h, so the paw volume observed after 2 h (i.e., from 0.2 mL to 0.42 mL) was considered as the disease state or starting point in this study. The improvement in the disease was reflected by a reduction in paw volume, which is usually indicated by calculating the % oedema inhibition. The percentage oedema inhibition was calculated for all groups by using the following formula [27,28].

% Oedema inhibition = 100 × [1 − (a − x)/(c − y)](7)

where ‘a’ and ‘x’ represent the paw volume after and before CG administration, respectively, at time ‘t’ and ‘c’ and ‘y’ represent the mean paw volume of control rats after and before CG administration, respectively, at time ‘t’.

#### 4.12.3. Determination of Inflammatory Markers

For all groups, the in vivo anti-inflammatory activity was terminated after 7 h from the initial induction of the rat paw oedema using CG injection. At this time point, the blood sample was withdrawn by the retro-orbital technique from each rat and transferred to a sterile glass vacuum tube (BIO-VAC) containing the gel and clot activator. The samples were centrifuged at 1000 rpm for 10 min. The serum was isolated for the measurement of inflammatory markers including CRP and IL-6. These inflammatory markers were measured by an Invitrogen Rat ELISA Kit (Sandwich ELISA Kit, Thermo Fisher Scientific, Waltham, MA, USA) with a pre-coated 96-well plate standard. The samples were placed into these wells. These samples were bound between an antibody pair (a specific antibody pre-coated and a second detector antibody), hence presenting a sandwich. The intensity of the signals produced by this complex was measured. The intensity represented the concentration of the CRP and IL-6 present in the sample [29].

#### 4.12.4. Histopathological Examination of Paw Tissues

At the end of the in vivo anti-inflammatory study, the rat paw tissues of all groups were removed and stored in formalin solution. The tissue samples were cut into thin slices by using a microtome. The sliced tissues were fixed on glass slides and stained with haematoxylin and eosin, and histopathological examination was performed under an optical microscope (Labomed, LA, USA) at 40× magnification [30].

### 4.13. Statistical Analysis

All the experiments were performed at least three times. The results were analyzed by using various statistical parameters. The results were expressed as the mean ± SD. The results were compared by using a t-test and two-way ANOVA, followed by Dunnett’s test. The software employed for this purpose included Microsoft Excel version 2016, SPSS (Statistical Package for Social Sciences) version 15.0 and GraphPAD Prism version 8.0. The results were considered significant at *p* ˂ 0.05.

## 5. Conclusions

Celecoxib-loaded single and dual crosslinked chitosan/guar gum-based hydrogel beads with desired features were fabricated. The prepared formulations released the loaded drug in a sustained manner. Single crosslinked beads released ~74% of the drug within 24 h, while in the case of the dual crosslinked formulation, only ~24% drug release was observed. The dual crosslinked formulation showed better mucoadhesion; however, single crosslinked hydrogel beads showed a higher swelling% and permeability. Moreover, the SC formulation showed a relatively better therapeutic performance in the rat paw oedema model. It was concluded that celecoxib-loaded crosslinked chitosan/guar gum-based hydrogel beads are capable of providing a sustained release of the drug and treating inflammatory conditions.

## Figures and Tables

**Table 1:** Mucoadhesion of SC5 and DC5 beads with goat intestinal mucosa.

Time (h)	1	2	3	4	5	6	8	10	12	24
% Adhesion SC5	100 ± 00	100 ± 00	100 ± 0.57	96 ± 0.55	88 ± 00	76 ± 0.57	64 ± 0.57	32 ± 00	20 ± 0.52	0 ± 0.5
% Adhesion DC5	100 ± 00	100 ± 00	100 ± 00	100 ± 00	100 ± 0.57	92 ± 0.57	84 ± 0.57	60 ± 00	48 ± 0.57	8 ± 00

**Table 2:** Different formulation and process parameters (variable and constant) of single and dual crosslinked hydrogel beads.

Variable Parameters				
	Formulation Code	CS (% *w*/*v*)	GG (% *w*/*v*)	
L	SC1	1.5 (−1)	10 (−1)	
	SC2	1.5 (−1)	30 (0)	
	SC3	1.5 (−1)	50 (1)	
M	SC4	2 (0)	10 (−1)	
	SC5	2 (0)	30 (0)	
	SC6	2 (0)	50 (1)	
H	SC7	2.5 (1)	10 (−1)	
	SC8	2.5 (1)	30 (0)	
	SC9	2.5 (1)	50 (1)	
L	DC1	1.5 (−1)	10 (−1)	
	DC2	1.5 (−1)	30 (0)	
	DC3	1.5 (−1)	50 (1)	
M	DC4	2 (0)	10 (−1)	
	DC5	2 (0)	30 (0)	
	DC6	2 (0)	50 (1)	
H	DC7	2.5 (1)	10 (−1)	
	DC8	2.5 (1)	30 (0)	
	DC9	2.5 (1)	50 (1)	
Constant Parameters (SC & DC) L (Low), M (Medium) & H (High)				
Total volume (polymer solution) (mL)	TPP 1% volume (mL)	Stirring speed (rpm)	GLA 2% volume (mL)	
			SC	DC
10	100	2000	0	10
where, for CS content (% *w*/*v*), −1 = 1.5, 0 = 2 and 1 = 2.5, and for GG content (% *w*/*v*), −1 = 10, 0 = 30 and 1 = 50				

**Table 3:** Rat groups detail with treatment protocols (*n* = 6).

Group	Codes	Description	CG (0.1 mL, 1%) Administration	Design of Treatment	Treatment Dose
1	NR	Normal (healthy)	No	No Treatment	0
2	NC	Negative Control	Yes	No Treatment	0
3	RF	Reference	Yes	Pure Celecoxib	15 mg/kg
4	PC	Positive Control	Yes	Celbex Capsule	15 mg/kg
5	SC	Single Crosslinked Hydrogel Beads	Yes	CX-loaded SC Hydrogel Beads	15 mg/kg
6	DC	Dual Crosslinked Hydrogel Beads	Yes	CX-loaded DC Hydrogel Beads	15 mg/kg

## Data Availability

The data are contained within the article.

## Abbreviations

Abbreviations	Full form/explanation
CS	Chitosan
GG	Guar gum
SC	Single crosslinked beads
DC	Dual/double crosslinked beads
EE%	Entrapment efficiency
LE%	Loading efficiency
CRP	C-reactive protein
IL-6	Interleukin-6
CX	Celecoxib
NSAID	Non-steroidal anti-inflammatory drug
GIT	Gastrointestinal tract
STPP/TPP	Sodium tripolyphosphate
GLA	Glutaraldehyde
PO_4_^−^	Phosphate group
NH_3_^+^	Amino group
-OH	Hydroxyl group
SEM	Scanning electron microscopy
FTIR	Fourier transform infrared spectroscopy
XRD	X-ray diffraction
DSC	Differential scanning calorimetry
TGA	Thermogravimetric analysis
Papp	Apparent permeability coefficient
CG	Carrageenan
S(EQ)	Equilibrium swelling ratio
